# Adherence and treatment patterns of disease-specific drugs among patients with pulmonary arterial hypertension: A nationwide, new-user cohort study

**DOI:** 10.3389/fphar.2022.1030693

**Published:** 2023-01-12

**Authors:** Cheng-Yu Tsai, Chuan-Wei Shen, Hsuan-Lin Lai, Chung-Yu Chen

**Affiliations:** ^1^ Master Program in Clinical Pharmacy, School of Pharmacy, Kaohsiung Medical University, Kaohsiung, Taiwan; ^2^ School of Pharmacy, Kaohsiung Medical University, Kaohsiung, Taiwan; ^3^ Division of Pharmacy, Zuoying Branch of Kaohsiung Armed Forces General Hospital, Kaohsiung, Taiwan; ^4^ Department of Pharmacy, Kaohsiung Medical University Hospital, Kaohsiung, Taiwan; ^5^ Department of Medical Research, Kaohsiung Medical University Hospital, Kaohsiung, Taiwan

**Keywords:** pulmonary arterial hypertension, treatment patterns, medication adherence, pharmacoepidemiogy, proportion of days covered

## Abstract

**Background:** Pulmonary arterial hypertension (PAH) is an incurable pulmonary disease that might result in right heart failure and death. Treatment guidelines recommend upfront or sequential combination therapy for patients with PAH. Recently, several PAH-targeted medications have been approved in Taiwan. This study aimed to investigate treatment patterns and medication adherence in real-world settings.

**Method:** This was a new-user design study on patients treated with PAH-specific medication between 1 January 2014, and 31 December 2019. Data were extracted from the National Health Insurance Research Database. Medication adherence was evaluated by the proportion of days covered (PDC). Adherence was defined as PDC ≥ .8. Statistical analyses were performed to compare the study outcomes. Logistic regression analysis was performed to identify the association between baseline characteristics and adherence. *P* < .05 indicated statistical significance.

**Results:** A total of 1,900 patients with PAH were identified, and 75.3% of them were females. The mean (standard deviation (SD)) age was 57.2 (17.5) years. Only 23 (1.2%) patients began the initial combination therapy. A total of 148 (7.8%) patients switched their initial treatment to another treatment, and 159 (8.4%) patients had sequential combination therapy. The most common combination therapy was endothelin receptor antagonist (ERA) plus phosphodiesterase-5 inhibitor (PDE5i), mostly macitentan plus sildenafil, for initial or sequential combination. The mean (SD) PDC was .71 (.33), and 1,117 (58.8%) patients were adherent. A significant difference in mean PDC was observed between initial ERA users and PDE5i users (*p* < .0001). No factor was significantly associated with medication adherence.

**Conclusion:** Patients with PAH mostly initiated sildenafil as monotherapy, and macitentan was added as a sequential combination therapy. The initial ERA and combination groups showed higher medication adherence. Further investigations are needed to identify other factors associated with adherence.

## Introduction

Pulmonary arterial hypertension (PAH) is a rare, progressive, and incurable pulmonary vascular disease characterized by pulmonary microvascular remodeling, resulting in higher pulmonary vascular resistance and right ventricular hypertrophy. This progressive condition can result in right heart failure and death if left untreated (Hassoun, 2021). In Taiwan, the estimated incidence of PAH was 501 cases per million from 1999 to 2011 (Chang et al., 2016). The treatment guidelines recommend oral monotherapy, including endothelin receptor antagonists (ERA), phosphodiesterase-5 inhibitors (PDE5i), and soluble guanylate cyclase stimulators (sGCs), or oral combination therapy for patients with low or intermediate risk and combination therapy, including intravenous prostacyclin analogs (PCA), for patients with high risk (Galiè et al., 2015a). Several PAH-specific medications have been approved and reimbursed for PAH by the Taiwan Food and Drug Administration and Taiwan National Health Insurance (TNHI) since 2015. However, utilization of these PAH-targeted treatments for patients with PAH is unclear in Taiwan. Furthermore, medication adherence and persistence are important issues for managing chronic diseases. Factors associated with PAH medication adherence remain controversial. Thus, this study aimed to investigate the treatment patterns and medication adherence of PAH-specific medications and identify any factors associated with medication adherence among patients with PAH.

## Materials and methods

### Study design and data source

This was a new-user design and 1-year follow-up cohort study. All data were extracted from the National Health Insurance Research Database (NHIRD), a single health insurance claims database covering more than 99.6% of the general population in Taiwan. The healthcare information in the database includes outpatients (emergency room utilization is part of the outpatient data), hospitalizations, dental services, and prescription drugs. The study period was from 1 January 2013, to 31 December 2020. This study was approved by the Institutional Review Board (IRB) of Kaohsiung Medical University Hospital (IRB number: KMUHIRB-E(I)-20220061).

### Study population

Patients with PAH were identified using 1) the International Classification of Diseases, 9th or 10th Revision, Clinical Modification (ICD-9/10-CM) diagnosis codes of pulmonary hypertension (PH) and diseases associated with PAH, 2) ICD-9/10-CM diagnosis codes of chronic thromboembolic pulmonary hypertension (CTEPH)-related conditions, and 3) TNHI claim codes of PAH-targeted medication. The first prescription date of any PAH-targeted medication was defined as the index date. The prescribed medication within 30 days after the index date was defined as the index treatment.

The inclusion criteria included patients with ≥1 claim for PAH-targeted medication between 1 January 2014, and 31 December 2019 (identification period), and patients with ≥1 claim for PH- or PAH-associated diseases within 6 months before or 1 month after the index date (eligibility period). The exclusion criteria included patients with ≥1 claim for CTEPH-related conditions (e.g., pulmonary embolism or deep vein thrombosis) within the eligibility period and their index treatment including riociguat, previous PAH-specific medication user before 1 January 2014 (washout period, a 1-year period where no PAH-targeted medication prescribed before the index date), patients with <2 claims for PAH-targeted medication during the follow-up period, age under 20 years on the index date, and missing data on gender or age. The follow-up period was censor, death, or 1 year after index date.

### Baseline characteristics

All data of sociodemographic information, including age, gender, level of National Health Insurance (NHI) premiums, urbanization level of residence, and residential area, were collected from the “Registry for Beneficiaries” dataset.

The patients’ genders were identified as either male or female. The exact age on the index date was calculated by subtracting the year of birth from the year of the index date. Patients were divided into three age groups (20–39, 40–64, and ≥65 years). NHI premiums were categorized into ten groups based on the patients’ monthly income. Patients with NHI premiums ≤22,800 (NTD) were classified as low income, and those >22,800 (NTD) were classified as high income according to the minimum wage in Taiwan (Chen et al., 2020).

More than 350 townships were categorized into a 7-level development stratification (Liu et al., 2006). Levels 1–2, 3–4, and 5–7 were classified as urban, suburban, and rural, respectively, according to a previous study (Cheng et al., 2019). The residential area of each patient was classified as northern, central, southern, and western areas.

The baseline comorbidities of PAH cohorts were collected from “Inpatient Expenditures by Admission” and “Ambulatory Care Expenditures by Visits” datasets and identified using the modified Elixhauser Comorbidity Index (Elixhauser et al., 1998; Moore et al., 2017). These comorbidities were defined as having ≥2 outpatient or ≥1 inpatient claims with ICD-9/10-CM diagnosis codes of these comorbidities within a year before and including the index date. The number of Elixhauser comorbidities was calculated and was categorized into three levels (0, 1-2, and ≥3).

The clinical examination records, including echocardiography, chest X-ray, and right heart catheterization (RHC), were collected from “Inpatient Expenditures by Admission” and “Ambulatory Care Expenditures by Visits” datasets and were identified using ICD-9-CM diagnosis codes, ICD-10 Procedure Coding System (ICD-10-PCS) codes, and TNHI claim codes (defined as having ≥1 claim within the eligibility period).

### Study outcomes

All the data on study outcomes were collected from the “Inpatient Expenditures by Admission,” “Ambulatory Care Expenditures by Visits,” “Details of Inpatient Orders,” and “Details of Ambulatory Care Orders” datasets.

Treatment with one category of PAH-targeted medication was defined as monotherapy, and treatment with two or more categories of PAH-targeted medication overlapping more than one day was defined as combination therapy. The index regimen was identified as the PAH-targeted medication used by the patients within 30 days after and including the index date. Treatment with other kinds of PAH-targeted medication different from the initial treatment was defined as switching. Any PAH-targeted medication added to the initial treatment was defined as a sequential combination. Stopping using PAH-targeted medication for more than 90 days (grace period) after running out of pills was defined as discontinuation. The 90-day interval for the treatment pattern of the Sankey diagram was based on the grace period. Day 0 indicates the initial/index treatment. Day 90, 180, and 270 indicates the first regimen within 90–179, 180–269, and 270–359 days after the index date.

Adherence to the initial monotherapy was evaluated using the proportion of days covered (PDC) calculated by dividing the number of days of available medication by the number of days of the follow-up period (Nau, 2012). This study presented two definitions of the denominator for PDC calculation. First, PDC1 was defined as the number of days of available medication divided by 365 days (1-year follow-up). Second, PDC2 was defined as the number of days of available medication divided by the number of days from the index date to the treatment modification, treatment discontinuation, or end of the last prescription (Prieto-Merino et al., 2021). PDC for the initial combination therapy was based on possession of any medication, which meant days covered by at least one medication in the regimen (Basak et al., 2014). PDC ≥ .8 was defined as adherent.

### Statistical analysis

Categorical variables were presented as numbers and percentages, whereas continuous variables were presented as means and standard deviations (SD) or median and interquartile range (IQR). Kruskal–Wallis H tests were performed to analyze PDC1 or PDC2 between the four index treatment groups, and the Chi-square or Fisher’s exact tests were performed to analyze the frequency of medical adherence (PDC≥.8 and PDC<.8) between four index group. Treatment patterns were analyzed descriptively and illustrated using Sankey diagrams. Logistic regression analysis was performed to assess the factors associated with adherence, and goodness of fit statistics for the logistic regression by C-statistic and Hosmer-Lemeshow test.

All statistical analyses were performed using SAS 9.4 (SAS Institute, Inc., Cary, North Carolina, United States). All the statistical analyses were two-tailed, and a *p*-value ≤ .05 was considered significant.

## Results

### Study population

**FIGURE 1:**
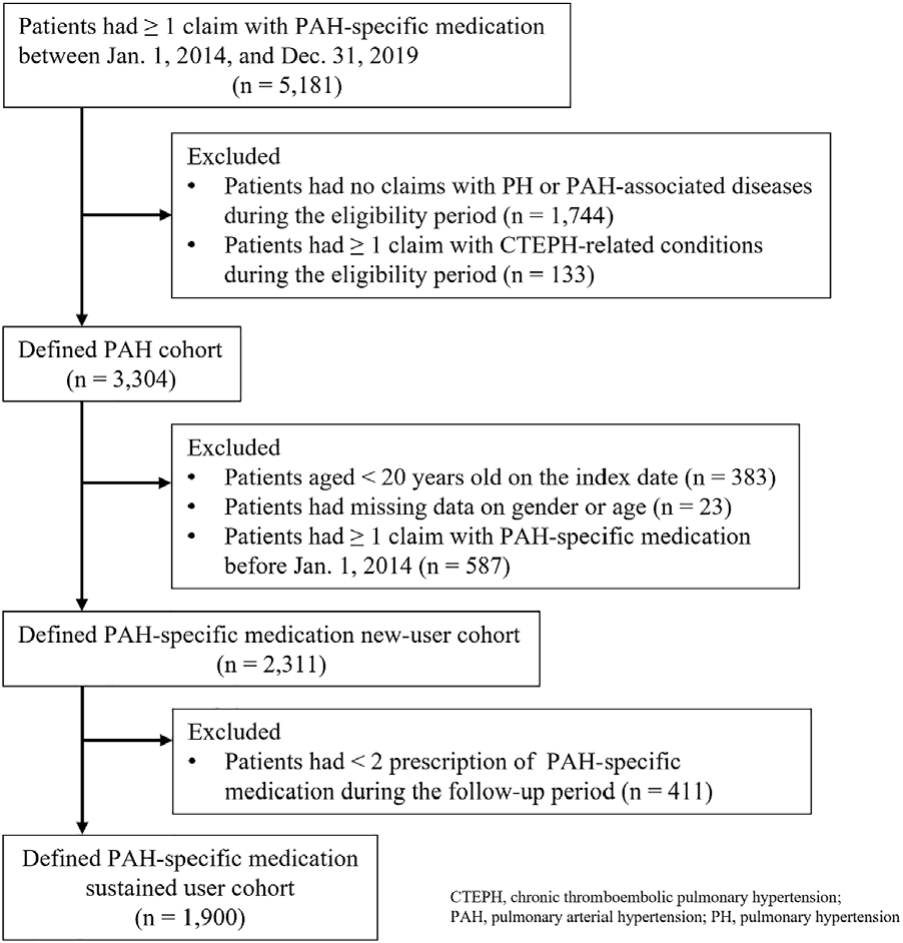
Study population.

A total of 5,181 patients were prescribed at least one PAH-specific medication between 1 January 2014, and 31 December 2019. After applying the exclusion criteria, 1,900 patients were involved in the analysis. Figure 1 shows the study population selection.

### Baseline characteristics and initial treatment

A total of 1,900 new and sustained users were included in the investigation of baseline characteristics. The mean (SD) age was 57.2 (17.5) years, with a predominance of female patients (*n =* 1,430; 75.3%). The percentage of patients aged 40–64 years was slightly higher than those aged ≥65 years (*n =* 839; 44.1% vs. *n =* 693; 35.5%). Most patients lived in the urban (*n =* 1,131; 59.5%) and northern areas (*n =* 882; 46.4%). The proportion of insurance premiums was similar between low-income and high-income groups (*n =* 949; 49.9% vs. *n =* 951; 50.1%). Over 90% of the patients had performed echocardiography (*n =* 1,867; 98.3%) and chest X-ray (*n =* 1,786; 94.0%), but only one-third of them had conducted RHC (*n =* 679; 35.7%). Most patients had 3 or more comorbidities (*n =* 1,205; 63.4%). Table 1 shows the baseline characteristics.

**TABLE 1:** Baseline characteristics and initial treatment.

Characteristics	Total (*n =* 1,900)
Female, n (%)	1,430 (75.3)
Age, mean (SD)	57.2 (17.5)
Age group, n (%)	
20–39	368 (19.4)
40–64	839 (44.1)
≥65	693 (35.5)
Insurance premium (NTD), n (%)	
>22,800	951 (50.1)
≤22,800	949 (49.9)
Urbanization, n (%)	
Urban	1,131 (59.5)
Suburban	580 (30.5)
Rural	189 (10.0)
Resident area, n (%)	
Northern area	882 (46.4)
Southern area	550 (29.0)
Central area	422 (22.2)
Eastern area	46 (2.4)
Examination, n (%)	
Echocardiography	1,867 (98.3)
Chest X-ray	1,786 (94.0)
Computed tomography	1,249 (65.7)
Right heart catheterization	679 (35.7)
Ventilation/perfusion lung scan	553 (29.1)
Magnetic resonance imaging	192 (10.1)
Elixhauser comorbidity index, n (%)	
None	57 (3.0)
1–2	638 (33.6)
≥3	1,205 (63.4)
Initial treatment, n (%)	
Monotherapy	1,877 (98.8)
Combination therapy	23 (1.2)
Monotherapy, n (% of initial monotherapy)	
ERA	82 (4.4)
PDE5i/sGCs	1,779 (94.8)
PCA	16 (.8)

ERA, endothelin receptor antagonist; PCA, prostacyclin analogues; PDE5i, phosphodiesterase-5 inhibitor; sGCs, soluble guanylate cyclase stimulator.

### Treatment patterns

Initial monotherapy was more common than initial combination therapy (*n =* 1,877; 98.8% vs. *n =* 23; 1.2%). Of the 1,877 patients with initial monotherapy, 94.8% (*n =* 1,779) received PDE5i/sGCs, followed by ERA and PCA (*n =* 82; 4.4% and *n =* 16; .8%). Moreover, sildenafil was the most popular medication prescribed as initial monotherapy (*n =* 1,764; 94.0%). During the 1-year follow-up period, the proportion of dual or triple combination therapy increased from 1.2% (*n =* 23) to 8.6% (*n =* 163).

**FIGURE 2:**
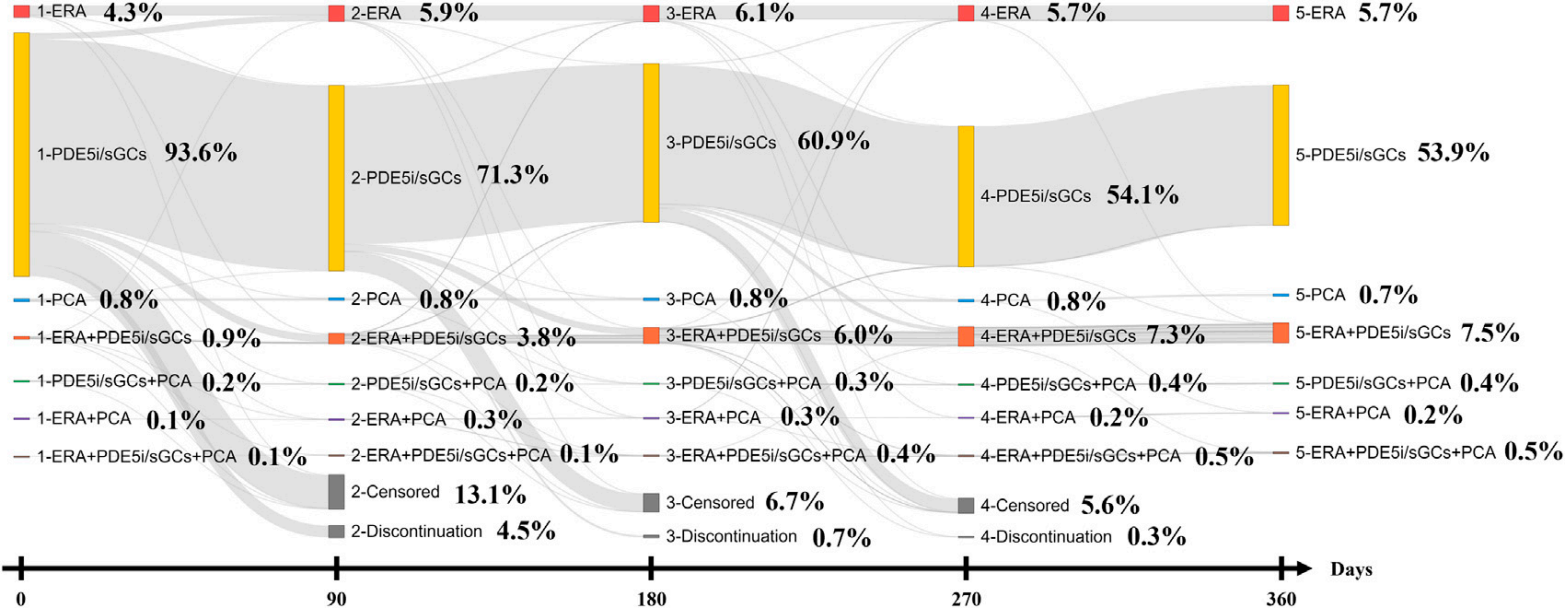
Treatment patterns of Sankey diagram (The 90 days interval for Sankey diagram was based on the grace period. Day 0 indicates the initial/index treatment. Day 90, 290, 270 indicates the first regimen within 90–179, 180–269, and 270–359 days after the index date).

Of those treated with ERA initially, 81.7% (*n =* 67) remained on the same regimen 90 days after the index date. Similar results were also observed in the initial PDE5i/sGCs treatment and initial PCA treatment groups (*n =* 1,349; 75.8% and *n =* 13; 81.3%). After one year of follow-up, more than a half of the patients remained initial treatment (ERA 69.5%, PDE5i/sGCs 57.1%, and PCA 68.8%, respectively). Figure 2 shows the treatment patterns from the index date to 365 days after the index date.

### Switching

Of the 1,900 patients, 148 (7.8%) changed their initial treatment. Of these patients, 84.5% (*n =* 125) were treated with sildenafil, and 52.7% (*n =* 78) switched medication from PDE5i to ERA monotherapy. Macitentan (*n =* 47; 31.8%) was the most popular ERA for switching, followed by bosentan (*n =* 30; 20.3%) and ambrisentan (*n =* 4; 2.7%).

### Sequential combination

Sequential combination therapy was typically dual therapy, with 142 (89.3%) patients being treated with ERA plus PDE5i combination. Only 7 (4.4%) patients had triple therapy as a second regimen.

### Proportion of days covered

The mean (SD) and median (IQR) of PDC1 for all patients were .71 (.33) and .89 (.45–.98), which were lower than those of PDC2 (.89 (.15), and .96 (.84–1.00)). Additionally, the percentage of adherent patients (PDC ≥.8) was 58.8% (*n =* 1,117) in PDC1, which was lower than that in PDC2 (*n =* 1,526; 80.3%). The goodness of fit of the logistic regression model showed fitting the data moderately well for PDC1 (c-statistic = .568; Hosmer-Lemeshow test *p* = .4405; Supplementary eTables S1, S2) and PDC 2 (c-statistic = .577; Hosmer-Lemeshow test *p* = .2651; Supplementary eTables S3, S4).

The mean (SD) of PDC1 and PDC2 of the initial ERA treatment group was significantly higher than that of the initial PDE5i/sGCs treatment group (.88 (.18) vs. .70 (.33); *p* < .0001 and .94 (.11) vs. .89 (.15); *p* = .0001). Besides, the mean (SD) of PDC2 of the initial combination group was significantly higher than that of the PDE5i/sGCs group (.96 (.06) vs. .89 (.15); *p* = .0156). Similar results were found in the percentage of adherent patients between initial treatment groups (*p* < .05) (Table 2).

**TABLE 2:** Proportion of days covered.

Variable	Total (*n =* 1,900)	Index treatment group				*p*-value
		ERA (*n =* 82)	PDE5i/sGCs (*n =* 1,779)	PCA (*n =* 16)	Combination (*n =* 23)	
PDC1, mean (SD)	.71 (.33)	.88 (.18)	.70 (.33)	.76 (.28)	.77 (.32)	<.0001
PDC1, median (IQR)	.89 (.45–.98)	.97 (.86–.99)	.87 (.43–.98)	.88 (.59–.98)	.92 (.81–.98)	
PDC2, mean (SD)	.89 (.15)	.94 (.11)	.89 (.15)	.86 (.21)	.96 (.06)	<.0001
PDC2, median (IQR)	.96 (.84–1.00)	.98 (.96–1.00)	.96 (.84–.99)	.98 (.78–1.00)	1.00 (.95–1.00)	
Medication adherence, n (%)						
PDC1 ≥0.8	1,117 (58.8)	65 (79.3)	1,024 (57.6)	10 (62.5)	18 (78.3)	.0003
PDC1 <0.8	783 (41.2)	17 (20.7)	755 (42.4)	6 (37.5)	5 (21.7)	
PDC2 ≥0.8	1,526 (80.3)	74 (90.2)	1,418 (79.7)	12 (75.0)	22 (95.7)	.0159
PDC2 <0.8	374 (19.7)	8 (9.8)	361 (20.3)	4 (25.0)	<3	

ERA, endothelin receptor antagonist; IQR, interquartile range; PCA, prostacyclin analogues; PDC, proportion of days covered; PDE5i, phosphodiesterase-5 inhibitor; SD, standard deviation; sGCs, soluble guanylate cyclase stimulator.

### Factors associated with medication adherence

The logistic regression analysis showed no significant association between good adherence (PDC1 ≥ .8) and baseline and clinical characteristics, including age (odds ratio (OR) .99; 95% CI .98–1.00; *p* = .1585), age group (OR 1.09; 95% CI .74–1.59; OR 1.02; 95% CI .53–1.93; *p* = .7538), gender (OR 1.12; 95% CI .90–1.39; *p* = .3044), index treatment (OR 2.66; 95% CI .97–7.29; *p* = .0571), diagnosis group (OR .91; 95% CI .75–1.12; *p* = .3765), comorbidity index (OR 1.39; 95% CI .80–2.42; OR 1.35; 95% CI .78–2.34; *p* = .5061), insurance premium (OR 1.12; 95% CI .93–1.35; *p* = .2276), urbanization level of residence (OR .85; 95% CI .60–1.20; OR .89; 95% CI .63–1.26; *p* = .6428), and residential area (OR .78; 95% CI .41–1.48; OR .59; 95% CI .31–1.12; OR .88; 95% CI .47–1.66). Furthermore, results showed no association between adherence with PDC2 and baseline and clinical characteristics (Table 3).

**TABLE 3:** Factor associated with the medication adherence.

Variables	PDC1 (*n =* 1,900)		PDC2 (*n =* 1,900)	
	OR (95% CI)	*p*-value	OR (95% CI)	*p*-value
Age	.99 (.98–1.00)	.1585	1.01 (1.00–1.03)	.1495
Age group				
20-39	Reference	0.7538	Reference	0.3535
40-64	1.09 (0.74-1.59)		0.88 (0.55-1.41)	
≥65	1.02 (0.53-1.93)		0.63 (0.29-1.40)	
Gender				
Male	Reference	0.3044	Reference	0.6506
Female	1.12 (0.90-1.39)		0.94 (0.72-1.23)	
Index treatment				
Monotherapy	Reference	0.0571	Reference	0.0628
Combination	2.66 (0.97-7.29)		6.78 (0.90-50.99)	
Diagnosis group				
PH only	Reference	0.3765	Reference	0.1192
aPAH	0.91 (0.75-1.12)		1.21 (0.95-1.55)	
Comorbidity index				
0	Reference	0.5061	Reference	0.3992
1-2	1.39 (0.80-2.42)		1.29 (0.66-2.55)	
≥3	1.35 (0.78-2.34)		1.09 (0.56-2.14)	
Insurance premium				
>22,800	1.12 (0.93-1.35)	0.2276	0.93 (0.74-1.17)	0.5221
≤22,800	Reference		Reference	
Urbanization				
Urban	0.85 (0.60-1.20)	0.6428	1.29 (0.86-1.94)	0.4093
Suburban	0.89 (0.63-1.26)		1.31 (0.87-1.98)	
Rural	Reference		Reference	
Resident area				
Northern area	0.78 (0.41-1.48)	0.0182	1.06 (0.50-2.22)	0.0071
Central area	0.59 (0.31-1.12)		0.75 (0.36-1.58)	
Southern area	0.88 (0.47-1.66)		1.33 (0.63-2.79)	
Eastern area	Reference		Reference	

aPAH, pulmonary arterial hypertension associated diseases; OR, odds ratio; PDC, proportion of days covered; PH, pulmonary hypertension.

## Discussion

This new-user design and 1-year follow-up cohort study analyzed a nationwide and population-based claims database in Taiwan to investigate the sociodemographic and clinical characteristics among patients with PAH and evaluate the treatment patterns, medication adherence, and persistence of PAH-specific medications in real-world settings.

This study showed that most patients initiated PDE5i/sGCs as monotherapy, especially sildenafil, which is consistent with findings reported by Burger et al. (2018), Studer et al. (2019), and Studer et al. (2020) (Burger et al., 2018; Studer et al., 2019; Studer et al., 2020). However, a study from the Japanese registry revealed 31.5% of 108 treatment-naїve patients received upfront dual or triple combination therapy. The higher proportion of index combination therapy might result from 65.7% of patients classified as World Health Organization functional class (WHO-FC) III-IV, and 41.2% of index combination therapy included intravenous epoprostenol (Tamura et al., 2017).

Upfront combination therapy was recommended based on the positive results from the AMBITION trial. This trial demonstrated that the combination of ambrisentan and tadalafil significantly reduced the time to composite clinical failure compared with monotherapy (hazard ratio (HR), .50; 95% CI, .35–.72; *p* < .001) (Galiè et al., 2015b). However, differences in initial treatment for patients with PAH were observed between the guideline recommendations and the findings of this study and other studies. Wissmüller et al. (2022) reported five main reasons for taking a single medication among 182 patients managed at experienced PH care centers, including 1) failure to escalate due to intolerability, 2) being at low risk and having a favorable response to monotherapy, 3) having mild PAH syndrome, 4) being older and having multiple comorbidities, and 5) lack of satisfactory evidence of combination therapy for some subgroups of PAH (HIV, portal hypertension, and CHD) (Wissmüller et al., 2022). In this study, the mean (SD) age of patients with PAH was 57.5 (17.8) years, and only one-fifth of them were aged <40 years. Moreover, 64.3% of these patients had three or more comorbidities that might reduce the attempts to initiate combination therapy. Another reason is related to the predominance of initial monotherapy in Taiwan. Unlike most PAH-specific medications, physicians do not need prior authorization to prescribe sildenafil for patients with PAH. Furthermore, the combination therapy was not recommended by TNHI unless the disease progressed or the treatment outcome failed. Our findings revealed that whether to initiate monotherapy or combination therapy depended on the disease severity and the local reimbursement rule.

This study investigated the second regimen for those who had treatment modifications during the one-year follow-up period and reported 307 (16.2%) patients changed their initial treatment. Over half of the patients (51.8%) added another PAH-specific medication to the index regimen. Most of the regimens were ERA plus PDE5i (96.9%), especially macitentan plus sildenafil (47.8%), followed by bosentan plus sildenafil (32.1%). The results are aligned with the guideline’s recommendation of sequential combination therapy and are similar to the findings from US databases (Burger et al., 2018; Studer et al., 2019; Studer et al., 2020).

Several randomized controlled trials (RCTs) have been conducted to evaluate the difference in long-term event-free survival as the primary outcome between combination therapy and monotherapy (Pulido et al., 2013; Galiè et al., 2015b; McLaughlin et al., 2015; Sitbon et al., 2015). A meta-analysis of those RCTs demonstrated a significant risk reduction for clinical worsening among patients treated with combination therapy compared with monotherapy (risk ratio (RR) .65; 95% CI .58–.72; *p* < .00001) (Lajoie et al., 2016). This finding supported sequential combination therapy for patients with PAH treated with monotherapy. Although several network meta-analysis studies revealed inconsistent conclusions about which monotherapy or combination therapy was the best regimen, ERA and PDE5i combination therapy might be one of the better options (Lin et al., 2018; Wang et al., 2018; Petrovič and Locatelli, 2020; Fu et al., 2021).

The SERAPHIN trial investigated the long-term composite outcomes of patients adding macitentan to the baseline treatment regimen, including sildenafil, and observed a significant risk reduction in the macitentan group (HR .55; 97.5% CI .39–.76; *p* < .001) (Pulido et al., 2013). A meta-analysis focusing on the effectiveness of ERA plus PDE5i combination, including bosentan plus sildenafil, reported that combination therapy significantly improved outcomes, including 6-minute walking distance (mean difference 15.64; 95% CI 2.67–28.61; *p* = .02) and time to clinical worsening (OR .56; 95% CI .41–.76; *p* = .0002), compared with monotherapy (Kirtania et al., 2019).

In this study, no significant association between adherence and baseline or clinical characteristics was found by logistic regression analysis. This finding is consistent with several studies that reported that age, gender, and socioeconomic status were not associated with medication adherence (Waxman et al., 2013; Kjellström et al., 2020). A previous study used the 8-item Morisky Medication Adherence Scale (MMAS-8) questionnaire to evaluate the factors and reported that older age, single-use medication, and higher comorbidities or co-medication had a positive association with adherence (Grady et al., 2018). However, the internal reliability testing showed that Cronbach’s alpha coefficient was .53 (less than the acceptable cut-off value of .7).

Although no significant factors were reported in this study, some differences were found between the initial treatment groups in PDC1 and PDC2. Several reasons should be considered to understand the difference between groups. First, the approval frequencies of medication administration for PAH were different between ERA and PDE5i/sGCs. The frequencies for the ERA group were ambrisentan QD, bosentan BID, and macitentan QD, while those for the PDE5i/sGCs group were sildenafil TID, tadalafil QD, and riociguat TID (Galiè et al., 2015a; Narechania et al., 2020). Since tadalafil was not approved in Taiwan, the medication administration frequency was lower in the ERA group than in the PDE5i/sGCs group (Burks et al., 2018). Although the impact of medication administration frequency on adherence between ERA and PDE5i/sGCs was not investigated, a study focusing on the factors associated with adherence in PDE5i users showed that tadalafil users had better adherence than sildenafil users due to the simpler frequency of medication administration (OR 2.59; 95% CI 1.60–4.22) (Waxman et al., 2013). Moreover, Grady et al. reported that higher administration frequency had a negative impact on medication adherence among patients with PAH (OR .39; 95% CI .24–.65) (Grady et al., 2018). Our study found higher medication adherence due to the lower administration frequency in the ERA group. Second, better clinical outcomes make patients more willing to take medication regularly (Burks et al., 2018). Besides, several meta-analyses demonstrated that upfront or sequential combination therapy improved clinical outcomes, such as 6-minute walking distance 6 MWD and time to clinical worsening, compared to monotherapy (Zhu et al., 2012; Lajoie et al., 2016; Pan et al., 2018; Kirtania et al., 2019). Third, those who had upfront or sequential combination therapy for PAH might have more severe disease status, such as WHO-FC III-IV, at the beginning or have disease progression. Thus, with increased awareness of the severity of the disease or knowledge of the disease itself, patients are more likely to take medication on demand and cooperate with the healthcare team (Burks et al., 2018; Ivarsson et al., 2018).

### Strengths and limitations

To the best of our knowledge, this research is the first population-based cohort study investigating PAH-specific medication prescribing patterns and evaluating medication adherence and persistence among patients with PAH in Taiwan.

The study population was identified from the NHIRD. All patients treated with PAH-targeted medication will be enrolled in the database. Namely, the findings from the current study could represent the clinical practice of PAH treatment in real-world settings.

However, this study has some limitations. First, the examination results, including echocardiography or RHC, were unavailable to confirm the diagnosis of PAH in the NHIRD. To ascertain the true diagnosis of patients with PAH, not only ICD-9/10-CM codes of PH- or PAH-associated diseases and NHI claim codes of PAH-specific medication were used, but also the specialty of the physician or enrollment of catastrophic illness registry were identified. The identification algorithm was validated by a previous study with a positive predictive value of 32.5–78.0% (Sprecher et al., 2020; Gillmeyer et al., 2021). Additionally, physicians need prior authorization to prescribe PAH-targeted medication, which means that patients’ medical charts would be reviewed before using most PAH-specific medications, except for sildenafil. Second, several baseline or clinical characteristics, such as smoking history, drinking habit, education status, hemodynamic data, and PH severity, could not be identified from the NHIRD. Thus, the association between medication adherence and these factors were not analyzed.

## Conclusion

This study provided an overview of patients with PAH who were newly treated with PAH-specific medication and showed that most patients were aged 40–64 years, with a dominance of females. Almost all patients (98.8%) initiated monotherapy, and sildenafil was mostly prescribed. Only one-fifth of the patients underwent treatment switching or sequential combination, while the treatment regimen did not change in most patients at the end of the follow-up period. Of those who underwent sequential combination, nearly 90% were treated with ERA plus PDE5i combination.

The PDC was .89, and the adherent rate was 80.3%. Medication adherence was higher in the index ERA and combination groups than in the PDE5i/sGCs group. No factor was significantly associated with the proportion of adherent patients. Thus, further comprehensive investigations are needed to identify any other factors associated with adherent PAH patients.

## Data Availability

The original contributions presented in the study are included in the article/Supplementary Material, further inquiries can be directed to the corresponding authors.
