# Supplementary material for: Adherence and treatment patterns of disease-specific drugs among patients with pulmonary arterial hypertension: A nationwide, new-user cohort study
Source: Front Pharmacol. 2023 Jan 12;13:1030693. doi: 10.3389/fphar.2022.1030693 (PMC9877219; doi:10.3389/fphar.2022.1030693)
Supplement: Supplementary file 1 [file DataSheet1.PDF]

Adherence and treatment patterns of disease-specific drugs among patients with pulmonary arterial hypertension: a nationwide, new-user cohort study

etable1 Goodness of fit of logistic regression model for PDC1

Association of Predicted Probabilities and Observed Responses			
Percent Concordant	56.8	Somers' D	0.136
Percent Discordant	43.2	Gamma	0.136
Percent Tied	0	Tau-a	0.066
Pairs	874611	c	0.568

etable2 Hosmer and Lemeshow goodness of fit test of logistic regression model for PDC1

Chi-Square	DF	p-value
7.9281	8	0.4405

etable3 Goodness of fit of logistic regression model for PDC2

Association of Predicted Probabilities and Observed Responses			
Percent Concordant	57.6	Somers' D	0.153
Percent Discordant	42.3	Gamma	0.153
Percent Tied	0	Tau-a	0.048
Pairs	570724	c	0.577

etable4 Hosmer and Lemeshow goodness of fit test of logistic regression model for PDC2

Chi-Square	DF	p-value
9.9988	8	0.2651
